# Whole-Grain *Oryza sativa* L. Flour Extract Exhibits Potent Antioxidant and Anti-Inflammatory Activity in Rats with Experimentally Induced Inflammation

**DOI:** 10.3390/molecules31061012

**Published:** 2026-03-18

**Authors:** Ioana Ferențiu, Tiberia Ioana Pop, Alina Elena Pârvu, Meda Sandra Orăsan, Dinu Bolunduț, Marcel Pârvu, Florica Ranga, Ciprian Ovidiu Dalai, Mădălina Țicolea, Anca Elena But, Lia Oxana Usatiuc, Raluca Maria Pop

**Affiliations:** 1 Pathophysiology, Department of Morphofunctional Sciences, Faculty of Medicine, “Iuliu Hatieganu” University of Medicine and Pharmacy, 400012 Cluj-Napoca, Romania; abrudan.ferentiu.ioana@elearn.umfcluj.ro (I.F.); orasan.meda@umfcluj.ro (M.S.O.); bolundut_dinu@elearn.umfcluj.ro (D.B.); madalinaticolea@umfcluj.ro (M.Ț.); ancabut@umfcluj.ro (A.E.B.); lia.usatiuc@umfcluj.ro (L.O.U.); 2 Department of Technical and Soil Sciences, Faculty of Agriculture, University of Agricultural Sciences and Veterinary Medicine, 400372 Cluj-Napoca, Romania; 3 Department of Medical Oncology, “Ion Chiricuță” Institute of Oncology, 400015 Cluj-Napoca, Romania; 4 Department of Biology, Babeș-Bolyai University, 400015 Cluj-Napoca, Romania; marcel.parvu@ubbcluj.ro; 5 Food Science and Technology, Department of Food Science, University of Agricultural Science and Veterinary Medicine, 400372 Cluj-Napoca, Romania; florica.ranga@usamvcluj.ro; 6 Department of Dental Medicine, Faculty of Medicine and Pharmacy, University of Oradea, 410073 Oradea, Romania; cipridalai@gmail.com; 7 Pharmacology, Toxicology and Clinical Pharmacology, Department of Morphofunctional Sciences, Faculty of Medicine, “Iuliu Hatieganu” University of Medicine and Pharmacy, 400012 Cluj-Napoca, Romania; raluca.pop@umfcluj.ro

**Keywords:** anti-inflammatory, antioxidant, *Oryza sativa* L., polyphenol, whole grain extract

## Abstract

Whole-grain rice (*Oryza sativa* L.) is a rich source of polyphenols. The *in vivo* mechanisms linking its phytochemical profile to antioxidant and anti-inflammatory effects remain incompletely defined. This study investigated the antioxidant and anti-inflammatory activity of a whole-grain rice flour 70% ethanol extract (OSEE) and correlated these effects with its phenolic composition. OSEE showed high total phenolic content 0.121 ± 0.002 mg GAE/g d.w.), a lower total flavonoid content (61.83 ± 4.03 µg QE/g d.w.), and a phenolic profile dominated by phenolic acids (~87%), with ferulic and protocatechuic acids among the most abundant constituents. OSEE displayed significant *in vitro* antioxidant activity in DPPH, FRAP, hydrogen peroxide, and nitric oxide scavenging assays. *In vivo* activity was evaluated in male Wistar rats with turpentine oil-induced acute inflammation using both therapeutic (post-induction) and prophylactic (pre-induction) protocols, testing three oral doses of lyophilized extract (1.0, 0.50, and 0.25 g/kg/day). *In vivo*, OSEE attenuated systemic oxidative stress (TOS, TAC, OSI, AOPP, MDA, NOx, 3-nitrotyrosine, total thiols) and the expression of pro-inflammatory markers (NF-κB p65, IL-1β, IL-18, caspase-1) in a dose-dependent manner with both protocols, with the highest dose producing the most consistent reductions, while the expression level of the anti-inflammatory factor IL-10 remained unchanged. PCA supported a shift in biomarker networks toward a non-inflamed profile. These findings indicate that OSEE exerts coordinated antioxidant and anti-inflammatory effects *in vivo* that are strongly associated with its phenolic composition.

## 1. Introduction

Rice (*Oryza sativa* L.) is a cereal crop of major global importance and a staple food for billions of people worldwide. Thousands of cultivars exist, differing in grain size, shape, and color; based on pigmentation, rice is commonly grouped into black, red, brown, and white varieties. Pigmented rice has long been consumed across Asia, and black rice has historically been viewed as a premium grain with perceived health benefits [1,2,3].

Brown rice or whole grain rice is the version of the unpolished grain. The bran layers of the rice caryopsis (sub-aleurone/aleurone, seed coat, and pericarp) concentrate dietary fiber, proteins, vitamins, minerals, and diverse phytochemicals, including phenolic acids and phytic acid. Milling removes the bran and germ and yields white rice with reduced fiber and micronutrient contents, whereas brown rice retains these tissues and typically has a lower glycemic index. Black rice is gluten-free and has been reported to contain bioactive constituents (e.g., essential amino acids and lipid-derived compounds), making it a potential option for gluten-restricted diets [2,3,4,5].

Polyphenols present in whole grains can modulate inflammatory signaling, and several rice-derived phenolics have demonstrated antioxidant and anti-inflammatory activity in experimental systems [3,6,7].

Oxidative stress arises when the formation of reactive species, including reactive oxygen and reactive nitrogen species (ROS/RNS), exceeds the capacity of the body’s intrinsic antioxidant defenses. Under normal conditions, enzymes such as superoxide dismutase, catalase, and glutathione peroxidase help control ROS levels and preserve redox balance. When this balance is disrupted, excessive ROS can damage cellular components, particularly membrane lipids, proteins, and nucleic acids. Nitric oxide is a gaseous, lipid-soluble signaling radical that usually supports cardiovascular health by helping regulate vascular tone, reducing platelet aggregation, and limiting smooth muscle cell proliferation. In an oxidative environment, however, nitric oxide may shift from a protective mediator to a harmful contributor to tissue injury. As a result, oxidative stress-related cellular damage is closely associated with the initiation and amplification of inflammatory responses [2,3,4,5].

Inflammation is a fundamental defense mechanism of the innate immune system that occurs when tissues are injured or invaded by pathogens. In the short term, this response is necessary for protection and tissue repair. However, when it becomes persistent or poorly regulated, it can contribute to severe tissue injury, chronic inflammatory disease, autoimmunity, fibrosis, cancer development, and ultimately organ dysfunction. Chronic inflammation may be worsened by unhealthy eating patterns, psychological stress, physical inactivity, and toxic environmental exposures. For this reason, current nutrition strategies increasingly highlight anti-inflammatory dietary patterns. These patterns typically prioritize whole grains, fruits, vegetables, and unsaturated fats, and rice is increasingly being considered a potentially useful component. Evidence from studies on whole grains suggests that dietary patterns high in fiber, resistant starch, and antioxidant compounds are linked to reduced systemic inflammatory markers. In addition, research on gluten-free diets indicates improvements in inflammatory biomarkers among people with celiac disease and other autoimmune disorders. Therefore, limiting early pro-inflammatory activation is considered a plausible therapeutic strategy [3,4,6,7].

Whole-grain rice has also been linked to multiple bioactivities in preclinical research, including effects relevant to neuroprotection, blood pressure, glucose metabolism, cancer biology, and antimicrobial defense [4,8]. The *in vivo* mechanisms linking its phytochemical profile to antioxidant and anti-inflammatory effects remain incompletely defined. Therefore, in this study, we evaluated *in vivo* the antioxidant and anti-inflammatory potential of a whole-grain *Oryza sativa* L. flour ethanol extract (OSEE) and characterized its major phytochemicals. Although rice has been proposed to influence inflammatory pathways, the mechanisms remain incompletely defined; thus, we examined oxidative stress indices and inflammasome-related mediators in an acute experimental inflammation model.

## 2. Results

### 2.1. Phytochemical Analysis

The total phenolic content (TPC) of OSEE estimated by the Folin–Ciocâlteu assay was 0.121 ± 0.002 mg GAE/g d.w. OSEE (GAE—gallic acid equivalent), and the total flavonoid content (TFC) was 61.83 ± 4.03 µg QE/g d.w. OSEE (QE—quercetin equivalent).

**Figure 1:**
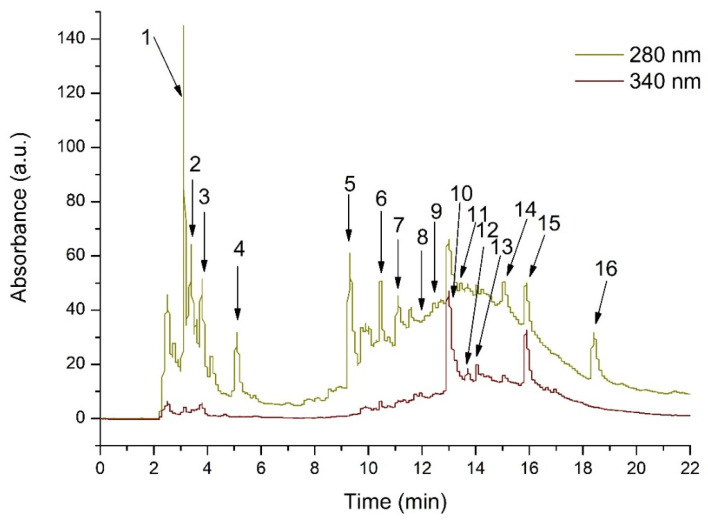
HPLC chromatogram of phenolic compounds from whole-grain *Oryza sativa* L. flour ethanol extract, measured at 280 and 340 nm. The peak identification is provided in Table 2.

HPLC analysis indicated that the phenolic profile of the OSEE was dominated by phenolic acids (approximately 87% of the quantified phenolics: hydroxybenzoic acids 58%, hydroxycinnamic acids 29%). Flavonoids accounted for about 13% of the quantified phenolics. Details are shown in Figure 1 and Table 1.

**Table 2:** *In vitro* antioxidant activity of the whole-grain *Oryza sativa* L. flour ethanol extract.

Sample	DPPH (μg TE/g d.w. Plant Material)	FRAP (μg TE/g d.w. Plant Material)	H_2_O_2_-Scavenging Activity (mg TE/g d.w. Plant Material)	NO-Scavenging Activity (mg QE/g d.w. Plant Material)
Whole-grain *Oryza sativa* L. flour ethanol extract (1 g/1 mL)	335.04 ± 28.52	256.29 ± 2.08	247.03 ± 18.04	341.28 ± 25.98
*p*-value	0.001	0.001	0.001	0.001

Values are means ± SD (*n* = 3) of three technical replicates. DPPH—2,2-diphenyl-1-picrylhydrazyl radical scavenging capacity; FRAP—ferric reducing antioxidant power assay; H_2_O_2_—hydrogen peroxide-scavenging activity; NO—nitric oxide radical-scavenging assay; TE—Trolox; QE—quercetin.

### 2.2. In Vitro Antioxidant Activity

*In vitro* antioxidant capacity assessed by DPPH, FRAP, H_2_O_2_, and NO scavenging assays indicated significant antioxidant activity for OSEE. Compared with Trolox (TE), OSEE showed stronger effects in the DPPH, FRAP, and H_2_O_2_ assays (*p* < 0.001). NO scavenging was also significant relative to quercetin (*p* < 0.001) (Table 2).

### 2.3. In Vivo Antioxidant and Anti-Inflammatory Activity

#### 2.3.1. Therapeutic Plan Effects

In the therapeutic plan, turpentine-induced acute inflammation on day 1 was followed by 10 days of daily treatments as follows: INFL1 with tap water, DICLO with diclofenac 10 mg/kg b.w./day, TX with Trolox 50 mg/kg b.w./day, OSEE group R100 with 1 g/kg/day, OSEE group R50 with 0.50 g/kg/day, and OSEE group R25 with 0.25 g/kg/day.

Therapeutic dosing with OSEE improved oxidative-inflammatory disturbance after turpentine challenge in a dose-related manner. Relative to the inflammation group, all OSEE doses produced a decrease in TOS and OSI (*p* < 0.01). R50 had the best antioxidant effect, but there were no significant differences among the three doses (*p* > 0.05). Furthermore, the effect of R50 was as good as that of DICLO and TX (Table 3).

Inflammation increased lipids, protein oxidation (MDA, AOPP), NO synthesis, and 3-NT formation (*p* < 0.001). The lipid oxidation marker (MDA) was reduced by the OSEE treatments (*p* < 0.001) in a dose-dependent manner, with R100 having the strongest inhibitory effect. There were no significant differences among the effects of R100, R50, and R25 on MDA (*p* > 0.05). OSEE downregulated MDA as strongly as DICLO and TX did (*p* > 0.05) (Table 4).

AOPP, the protein oxidation marker, was increased in the INFL1 group (*p* < 0.001), and all three OSEE dilutions lowered AOPP. R50 had the best inhibitory effect, but there were no significant differences among the effects of the three OSEE dilutions (*p* > 0.05).

NO and 3-NT, the nitrosative-stress indicators, were also decreased by OSEE treatment. R100 and R25 had the best inhibitory activity on NO secretion and 3-NT formation (*p* < 0.001), and the effects were comparable to those of DICLO and TX (Table 4).

All OSEE treatments shifted antioxidant readouts (TAC and total thiols) toward control values in a dose-dependent manner, with R100 having the best antioxidant effect (*p* < 0.001). R50 and R25 showed smaller improvements (*p* < 0.01). OSEE dilutions had better antioxidant activity than DICLO, but lower antioxidant activity than TX (Table 3 and Table 4).

Turpentine-induced inflammation markedly increased the pro-inflammatory markers NF-κB, IL-1β, IL-18, and caspase-1. All OSEE doses reduced these mediators in a concentration-dependent fashion, with inhibition being strongest with R100 (*p* < 0.001). For R100, the NF-κB expression was lower than that seen in the control, DICLO, and TX groups (*p* < 0.001). IL-1β and IL-18 levels were lowered, though not to the level seen in the control group, in a dose-dependent manner, with the higher dose having the stronger inhibitory effect (*p* < 0.001). All OSEE dilutions reduced caspase-1 significantly (*p* < 0.001), to levels lower than those seen in the control and DICLO groups, but above those in the TX group. IL-10, the anti-inflammatory marker, did not differ across groups (*p* > 0.05) (Table 5).

Collectively, the therapeutic protocol results show that OSEE attenuates oxidative stress and inflammatory activation, with greater efficacy at higher doses.

**Figure 2:**
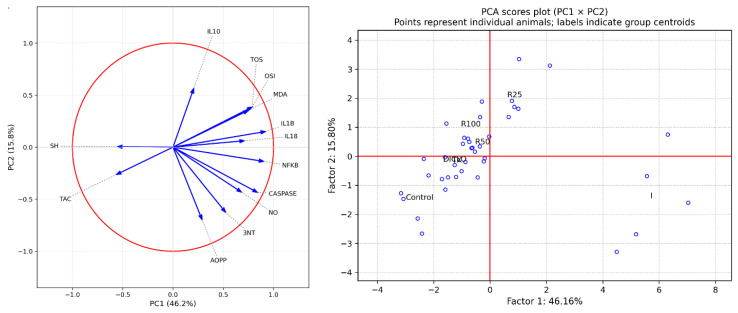
Principal component analysis (PCA) correlation circles of oxidative stress and inflammatory markers in the therapeutic plan. Left: Global PCA correlation circle (loading plot) for the therapeutic protocol (control, inflammation, diclofenac, Trolox, R100, R50, R25). Vectors represent variable loadings (correlations) with the first two principal components (PC1 and PC2), computed from the same pooled PCA solution used for the score plot. Vector direction indicates the association with each principal component, while vector length reflects the contribution of each biomarker to the respective component. The red circle denotes the unit correlation circle. Right: Global PCA score plot (PC1 × PC2) for the therapeutic protocol (control, inflammation, diclofenac, Trolox, R100, R50, R25). Each point represents an individual animal projected onto the first two principal components; labels indicate group centroids. The percentage of variance explained by each component is shown on the axes. PC1 and PC2 were derived from a single PCA computed on the pooled dataset (all groups combined), enabling direct comparison of group separation in the shared PCA space.

PCA was performed on the pooled therapeutic dataset to summarize the joint variation of oxidative stress-, inflammation-, and apoptosis-related biomarkers. Bartlett’s test supported dimensionality reduction (*p* < 0.001), and communalities indicated adequate representation of variables by the retained components. In the global solution, PC1 explained 46.2% of the variance, and PC2 explained 15.8% (cumulative 62.0%). The score plot (PC1 × PC2) showed clear separation of the inflammation group towards positive PC1 values, whereas the controls clustered on the negative side of PC1 (Figure 2).

The treated groups occupied intermediate positions, with R100 and R50 generally closer to the control PC1 region than R25. The global correlation circle for the same PCA space indicated that positive PC1 was driven mainly by oxidant and pro-inflammatory markers (TOS/OSI, MDA, IL-1β, IL-18, NF-κB, caspase activity), while antioxidant defenses (TAC and SH) projected in the opposite direction. PC2 captured additional variability related to protein oxidation, redox balance, and anti-inflammatory signaling. Collectively, the global PCA scores and loading plots support a dose-related multivariate shift of the biomarker profile away from the inflammatory pattern following extract administration (Figure 2).

#### 2.3.2. Prophylactic Plan Effects

For the prophylactic protocol, treatments were then administered for 10 days, INFL11 with tap water, TX with Trolox 50 mg/kg b.w./day, OSEE group R100 with 1 g/kg/day, OSEE group R50 with 0.50 g/kg/day, and OSEE group R25 with 0.25 g/kg/day, and acute inflammation was induced on day 11.

Inflammation induced on the 11th day strongly upregulated TOS and OSI (*p* < 0.001) and reduced TAC (*p* < 0.001). Pre-treatment with OSEE blunted the subsequent general oxidative stress markers; animals receiving the highest prophylactic dose (R100) exhibited significantly lowered TOS and OSI (*p* < 0.001) and increased TAC (*p* < 0.05) (Table 6).

In the inflammation group, oxidative damage and nitrosative stress markers (AOPP, MDA, NO, and 3-nitrotyrosine) were significantly upregulated by inflammation (*p* < 0.001). OSEE downregulated MDA and NO in a dose-dependent fashion, with the higher dose being the best inhibitor (*p* < 0.001). However, there was no significant difference among the three OSEE doses regarding their effects on MDA and NO (*p* > 0.05). For AOPP, R50 was the best inhibitor (*p* < 0.001), and for 3-NT, R25 had the best reduction activity (*p* < 0.001). The antioxidant capacity, evaluated by measuring SH, showed that R50 provided little antioxidant protection (*p* < 0.05), whereas R100 and R25 increased SH toward the control value (*p* < 0.01) (Table 7).

Consistent with these redox findings, inflammation strongly upregulated NFκB-p65, IL-1β, IL-18, and caspase-1 (*p* < 0.001). Prophylactic OSEE prevented this rise in a dose-dependent manner: for NFκB-p65 and caspase-1, R100 produced the most pronounced suppression (*p* < 0.001), but for IL-1β and IL-18, R25 yielded the most significant reductions (*p* < 0.001). When compared to TX, OSEE had a lower effect on NFκB-p65 and IL-1β. R50’s effect on IL-18 and caspase-1 was comparable to that of TX (*p* > 0.05). IL-10 remained unchanged (*p* > 0.05) (Table 8).

For the prophylactic protocol, principal component analysis (PCA) was used to summarize the multivariate relationships among oxidative stress and inflammatory markers.

**Figure 3:**
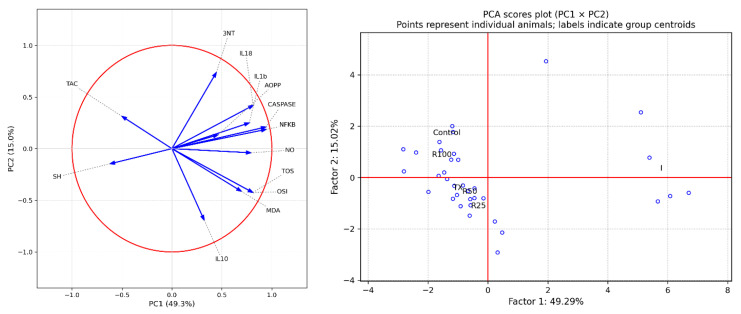
Principal component analysis (PCA) correlation circles of oxidative stress and inflammatory markers with the prophylactic plan. Left: Global PCA correlation circle (loadings plot) for the therapeutic protocol (control, inflammation, diclofenac, Trolox, R100, R50, R25). Vectors represent variable loadings (correlations) with the first two principal components (PC1 and PC2), computed from the same pooled PCA solution used for the score plot. Vector direction indicates the association with each principal component, while vector length reflects the contribution of each biomarker to the respective component. The red circle denotes the unit correlation circle. Right: Global PCA score plot (PC1 × PC2) for the therapeutic protocol (control, inflammation, Trolox, R100, R50, R25). Each point represents an individual animal projected onto the first two principal components; labels indicate group centroids. The percentage of variance explained by each component is shown on the axes. PC1 and PC2 were derived from a single PCA computed on the pooled dataset (all groups combined), enabling direct comparison of group separation in the shared PCA space.

Principal component analysis (PCA) was conducted to examine the multivariate structure of oxidative stress, inflammatory, and apoptotic biomarkers in the prophylactic protocol using all animals pooled across groups. The first two principal components accounted for the majority of the explained variance and were retained for visualization, in line with the scree-plot inflection after PC2. In this dataset, PC1 explained approximately half of the total variance, and PC2 contributed an additional ~15%, providing a two-dimensional representation capturing the dominant biomarker variability. Communalities indicated that the included variables were adequately represented by the extracted components. The global PCA score plot (PC1 × PC2) showed separation between the inflammation group and the prophylactically treated groups in the shared PCA space, with group centroids illustrating the overall shift in multivariate profiles. The corresponding global correlation circle (loading plot) indicated that the positive PC1 was primarily associated with oxidant and pro-inflammatory activities, whereas antioxidant-related parameters (TAC and SH) projected in the opposite direction, consistent with an oxidative–inflammatory gradient. PC2 captured additional variability linked to redox balance and nitrosative/protein oxidation markers (e.g., NO, 3-nitrotyrosine, AOPP), together with contributions from IL-10 (Figure 3). Taken together, the PCA visualizations support treatment-related multivariate shifts in the prophylactic setting, with increasing separation from the inflammatory profile at higher extract concentrations.

## 3. Discussion

Overall, our data indicate that OSEE exhibits meaningful antioxidant activity *in vitro* and reduces both oxidative stress and systemic inflammatory signaling *in vivo*, in association with its phenolic composition.

Diet is a modifiable determinant of chronic, low-grade inflammation, and shifts in food habits have been linked to higher burdens of inflammatory disorders and cancer. Consequently, there is strong interest in identifying natural dietary constituents that can counter these processes [3].

Polyphenols are among the most structurally diverse phytochemicals in plant foods, and epidemiological evidence links polyphenol-rich dietary patterns to a lower risk of major non-communicable diseases. Many of these compounds also display direct antioxidant and anti-inflammatory actions in experimental models [5].

The elevated total phenolic (TPC) and total flavonoid (TFC) contents of OSEE confirm that the extract is a relevant source of polyphenols. Reported differences among white, red, and black rice are largely explained by tissue distribution (bran vs. embryo/endosperm) and genotype; pigmented rice flours typically retain more bran, and therefore more phenolics. Our values are comparable with those described for other gluten-free grains, including teff [13,14,15].

Because antioxidant capacity often scales with total phytochemical load rather than any single molecule, we further characterized OSEE using HPLC-based profiling [16].

Previous work shows that whole-grain *O. sativa* is particularly rich in phenolic acids and flavonoids, including ferulic, *p*-coumaric, vanillic, gallic, syringic, and protocatechuic acids, together with flavonoids such as tricin, luteolin, and apigenin derivatives. The relative abundance of these compounds varies with cultivar, environment, processing, and storage [17,18,19].

Consistent with these reports, our HPLC-DAD-ESI-MS screening detected a broad spectrum of phenolics in OSEE. Phenolic acids predominated, with ferulic acid and protocatechuic acid showing the highest concentrations, while luteolin- and apigenin-glycosides were the main flavonoids. This profile aligns with prior analyses of pigmented rice extracts [1]. Polyphenols are detectable in blood and tissues at nanomolar to low micromolar concentrations and are found mainly as conjugated metabolites, especially glucuronides, which may also undergo methylation. As a result, their capacity to act as direct antioxidants is limited. Instead, their physiological relevance is thought to rely more on indirect mechanisms, such as triggering signaling pathways that upregulate genes involved in oxidative stress and inflammatory responses, including those encoding transcription factors and cytokines [20]. In addition, the biological effects of phenolic compounds may be driven by metabolites formed *in vivo*; recent evidence indicates that these metabolites can exhibit antioxidant and anti-inflammatory (anti-phlogistic) activities [21].

Given the high phenolic content, we anticipated strong radical-scavenging activity, which was supported by DPPH, FRAP, H_2_O_2_, and NO assays. The similarity between TPC and antioxidant readouts suggests that phenolics are key contributors to the *in vitro* activity [2,16,19].

*In vivo*, reactive oxygen species (ROS) can oxidize lipids, proteins, and nucleic acids. When ROS generation exceeds coordinated enzymatic and non-enzymatic defenses, oxidative stress develops; therefore, assessing a panel of oxidant and antioxidant biomarkers provides a more reliable picture than a single endpoint.

We first assessed overall redox status using TOS (oxidant load), TAC (antioxidant capacity), and the derived OSI. Inflammation increased TOS and OSI and lowered TAC, while OSEE counteracted these changes. In the therapeutic protocol, OSEE improved both oxidant and antioxidant indices, whereas in the prophylactic protocol, the dominant effect was a reduction in oxidant burden.

To complement these global indices, we quantified downstream products of protein and lipid oxidation, which integrate short-lived ROS activity over time and serve as practical markers of oxidative injury. AOPPs represent oxidatively modified proteins and are widely used as indicators of oxidative stress; they may also amplify inflammation by stimulating cytokine release [22]. Lipid peroxidation produces aldehydes such as MDA, a common marker of membrane oxidative damage [23]. OSEE reduced both AOPP and MDA, in some comparisons more strongly than diclofenac or Trolox, supporting an overall reduction in oxidative injury [6].

Nitric oxide (NO) is produced from L-arginine by nitric oxide synthases. During inflammatory activation, inducible NOS (iNOS) can markedly increase NO production. Because NO is rapidly converted to nitrite and nitrate, these stable products are commonly quantified [2,3,24,25]. OSEE lowered NO with both the prophylactic and the therapeutic regimens. Phenolic-rich black rice fractions, including ferulic acid, have been reported to suppress NF-κB signaling and downregulate iNOS expression. This mechanism could explain the reduced NO synthesis seen in our model too. By limiting concurrent NO and superoxide formation, the extract would also be expected to reduce reactive nitrogen species such as peroxynitrite and the downstream marker 3-nitrotyrosine, consistent with our findings [2,3].

Total thiols (SH) reflect the combined pool of free and protein-bound sulfhydryl groups, including glutathione and cysteine-containing proteins, and are often used as a functional readout of antioxidant reserves. OSEE produced a moderate increase in circulating thiols, which may reflect reduced oxidative consumption and/or contributions from thiol-containing constituents naturally present in cereal matrices [5].

Taken together with prior reports, these results support the view that whole-grain rice phenolics can mitigate oxidative stress across different experimental contexts.

Oxidative stress and inflammation reinforce each other and contribute to chronic conditions such as type 2 diabetes and cardiovascular disease. Plant-derived polyphenols may interrupt this loop through radical scavenging, metal chelation, and modulation of cellular signaling pathways [1,6,26].

Innate immune responses are initiated by pathogen-associated molecular patterns (PAMPs) and danger-associated molecular patterns (DAMPs), which promote leukocyte recruitment and macrophage activation. NF-κB is a central transcription factor that drives the expression of pro-inflammatory cytokines and enzymes such as COX-2 and iNOS [3,4]. In our model, turpentine increased NF-κB-p65, and OSEE reduced NFκB-p65 in both experimental protocols, although the effect was less pronounced than that of diclofenac or Trolox.

NF-κB activation can also prime inflammasome pathways. NLRP3 inflammasomes are cytosolic protein complexes composed of a sensor, the adaptor ASC, and pro-caspase-1; their assembly activates caspase-1, promotes maturation of IL-1β and IL-18, and can initiate pyroptosis [26]. Because dysregulated NLRP3 activation can sustain tissue injury and chronic inflammation, this pathway is considered a therapeutic target [27].

Multiple studies on pigmented rice extracts report anti-inflammatory effects that include suppression of NLRP3-related signaling and reduced IL-1β/IL-18 production, particularly for anthocyanin-rich fractions [27,28,29]. However, evaluations of whole-grain rice flour ethanol extracts as both preventive and therapeutic interventions remain limited.

IL-1β and IL-18 are synthesized as inactive precursors (pro forms) and require caspase-1 cleavage to generate active cytokines. Once released, these mediators can amplify inflammation through NF-κB activation and induction of additional cytokines [30,31,32,33].

As expected, turpentine-induced inflammation increased caspase-1, IL-1β, and IL-18. OSEE reduced all three markers, suggesting inhibition of inflammasome-associated activation. In contrast to some reports on isolated phenolics, IL-10 did not change during our short protocol [2,3].

In both *in vivo* protocols, oxidative stress and inflammation markers did not follow a traditional dose-response relationship. Some parameters exhibited a linear dose-dependent effect, but others had a non-linear effect. Compounds that interact with ROS, including many polyphenols, can participate in redox reactions in ways that are either protective or damaging, depending on conditions. In living systems, boosting the body’s own antioxidant enzyme defenses appears to matter more than relying on direct free-radical neutralization from dietary antioxidants [20,34]. Many plant-derived extracts—particularly those rich in polyphenols—have shown non-linear effects in human studies, where smaller or more diluted doses can sometimes lead to a greater overall improvement in oxidative stress biomarkers. One explanation is that polyphenols often do not function primarily as direct radical scavengers. Instead, at low to moderate intakes, they may act as mild biological stressors that trigger endogenous protective pathways and increase antioxidant capacity [34,35]. Furthermore, an antioxidant may shift toward pro-oxidant behavior based on several variables, including the availability of metal ions, its concentration within the surrounding matrix, and its intrinsic redox potential. For example, flavonoids such as quercetin and kaempferol have been observed to promote oxidative reactions in systems that contain transition metals, and phenolic compounds can become pro-oxidant in the presence of redox-active metals (e.g., iron or copper), which can drive redox cycling and generate phenoxyl radicals capable of harming lipids, proteins, and DNA [35].

The PCA results further support a coupled oxidative-inflammatory response: groups receiving OSEE shifted toward the control pattern, indicating concurrent normalization of redox and inflammatory variables.

This study has limitations. We used an acute inflammation model and no model of chronic experimental inflammation. A single ethanolic extract was tested, and no other solvents were used. We did not perform pharmacokinetic or dedicated toxicological analyses. Chronic models, comparisons with isolated constituents, and larger confirmatory experiments will be important to refine the mechanism and translational relevance.

## 4. Materials and Methods

### 4.1. Chemical Reagents

Folin–Ciocâlteu reagent, quercetin, gallic acid, standard chlorogenic acid (>98% HPLC), luteolin, and apigenin (>99% HPLC) were purchased from Sigma-Aldrich (St. Louis, MO, USA); acetonitrile of HPLC purity was obtained from Merck (Darmstadt, Germany); ultrapure water for the HPLC analysis was purified using a Direct-Q UV system from Millipore (Billerica, MA, USA); Trolox (6-hydroxy-2,5,7,8-tetramethylchroman-2-carboxylic acid) was obtained from Alfa-Aesar (Karlsruhe, Germany); ethanol, ferrous ammonium sulfate, hydrochloric acid, hydrogen peroxide (H_2_O_2_), methanol, *N*-(1-naphthyl) ethylenediaminedihydrochloric acid (NEDD), ortho dianisidine dihydrochloric acid (3-3′-dimethoxybenzidine), o-phthalaldehyde, thiobarbituric acid, sodium nitroprusside (SNP), sulphanilic acid, sulfanylamide (SULF), vanadium chloride (III) (VCl_3_), and xylenol orange [ocresosulfonphthalein-3,3-bis (sodium methyliminodiacetate)] were obtained from Merck (Darmstadt, Germany); ELISA kits (Elabscience Bionovation Inc. (Houston, TX, USA)) for rat NF-κB-p65 (E-EL-RO674), IL-1β (E-EL-0012), IL-18 (E-EL-R0567), caspase-1 (E-EL-R0371), and IL-10 (E-EL-R0016) were obtained from Elabscience Bionovation Inc. (Houston, TX, USA).

### 4.2. Plant Material Extract Preparation

Whole-grain *Oryza sativa* L. flour (Bauck GmbH, Rosche, Germany) was used as the starting material. A 70% (*v*/*v*) ethanol solvent was selected to recover both polar and moderately lipophilic constituents, and extraction was performed at room temperature using a modified Squibb cold repercolation procedure [36]. Lyophilized OSEE was stored at 4 °C until use, and the doses were prepared by diluting in 1 mL dH_2_O/animal daily.

### 4.3. Phytochemical Analysis

#### 4.3.1. Total Polyphenol Content

Total phenolic content (TPC) was quantified using a Folin–Ciocâlteu colorimetric assay adapted from Bhatti et al. [37]. Briefly, 2 mL of the OSEE solution was diluted 1:25. Folin–Ciocâlteu reagent (1 mL) and distilled water (10 mL) were added, and the volume was brought to 25 mL with sodium carbonate solution (290 g/L). After a 30 min incubation in the dark, the absorbance was read at 760 nm. TPC was calculated from a gallic acid calibration curve (10–500 µg/mL; R^2^ = 0.999) and expressed as mg gallic acid equivalents per g dry weight (mg GAE/g d.w.).

#### 4.3.2. Total Flavonoid Content

Total flavonoid content (TFC) was determined as previously described [37]. To 1 mL of OSEE solution, 0.3 mL of 5% NaNO_2_ was added, followed by 0.3 mL of 10% AlCl_3_. Next, 2 mL of 1 M NaOH was added, and the final volume was adjusted to 10 mL with distilled water. After 15 min, the absorbance was measured at 510 nm. TFC was calculated using a quercetin standard curve (1–50 µg/mL; R^2^ = 0.999) and expressed as mg quercetin equivalents per 100 g dry weight (µg QE/100 g d.w.).

#### 4.3.3. High-Performance Liquid Chromatography Coupled with Electrospray Ionization Mass Spectrometry (HPLC-ESI MS) Analysis

Phenolic profiling was performed by HPLC-DAD coupled to single-quadrupole MS (Agilent 1200 HPLC Agilent Technologies Inc., Santa Clara, CA, USA)with DAD; Agilent 6110 MS). Separation was performed using an Eclipse XDB-C18 (Agilent Technologies Inc., Santa Clara, CA, USA) column (4.6 × 150 mm, 5 µm) at room temperature. Mobile phase A was 0.1% acetic acid in water–acetonitrile (99:1, *v*/*v*), and mobile phase B was 0.1% acetic acid in acetonitrile. The gradient was 95% A (0–2 min), 95–60% A (2–18 min), 60–10% A (18–20 min), 10% A (20–24 min), followed by a return to 95% A in 1 min and a hold for 5 min. The flow rate was 0.5 mL/min; DAD chromatograms were collected at 280 nm (phenolic acids) and 340 nm (flavonoids). MS detection was performed by ESI in positive mode (350 °C, 3000 V; nitrogen 8 L/min), with scanning from 100 to 1000 *m*/*z*. Compounds were tentatively assigned based on their UV–Vis spectral characteristics, mass spectral data, comparison with authentic standards analyzed under identical chromatographic conditions, and previously reported data. For quantification, the lyophilized extract was dissolved in MeOH, and external calibration curves were used: chlorogenic acid for phenolic acids (R^2^ = 0.9937; LOD 0.41 µg/mL; LOQ 1.64 µg/mL), luteolin for flavones (R^2^ = 0.9972; LOD 0.26 µg/mL; LOQ 0.95 µg/mL), and rutin for flavonols (R^2^ = 0.9981; LOD 0.21 µg/mL; LOQ 0.84 µg/mL) [28].

### 4.4. In Vitro Antioxidant Activity Analysis

#### 4.4.1. 2,2-Diphenyl-1-picrylhydrazyl (DPPH) Radical Scavenging Capacity

DPPH radical scavenging was evaluated using a standard method [37]. Three milliliters of OSEE solution were mixed with 1 mL of 0.1 mM DPPH in methanol and incubated for 30 min in the dark at room temperature. Absorbance was recorded at 517 nm, and activity was calculated as AA% = [(A control − A sample)/A control] × 100. A Trolox calibration curve (0.5–5 µg/mL; R^2^ = 0.986) was used to express results as µg Trolox equivalents per g dry weight (µg TE/g d.w.), and IC50 values were derived accordingly.

#### 4.4.2. Ferric Reducing Antioxidant Power (FRAP) Assay

Ferric reducing antioxidant power (FRAP) was measured as previously described [38]. An aliquot of 100 µL OSEE solution was added to 3.4 mL FRAP reagent. After 30 min, the absorbance was read at 593 nm. The results were calculated from a Trolox standard curve (25–200 mg/mL; R^2^ = 0.999) and reported as µg Trolox equivalents per g dry weight (µg TE/g d.w.).

#### 4.4.3. Hydrogen Peroxide (H_2_O_2_)-Scavenging Activity

Hydrogen peroxide-scavenging capacity was assessed according to a published procedure [38]. OSEE solution was added to an H_2_O_2_ solution, and after 10 min, the absorbance at 230 nm was measured against phosphate buffer. Scavenging (%) was computed as (A control − A sample)/A control × 100, and IC50 values were expressed as mg Trolox equivalents per g dry weight (mg TE/g d.w.) using a Trolox calibration curve (10–100 µg/mL; R^2^ = 0.999).

#### 4.4.4. Nitric Oxide (NO) Radical-Scavenging Assay

NO radical scavenging was determined using sodium nitroprusside (SNP) as the NO donor [38]. OSEE solution (0.5 mL) was combined with an SNP mixture (2 mL SNP + 0.5 mL PBS, pH 7.4) and incubated for 2.5 h at 25 °C. Then, 0.5 mL of the reaction mixture was mixed with 1 mL sulphanilic acid; after 5 min, 1 mL *N*-(1-naphthyl)ethylenediamine dihydrochloride was added. After 30 min in the dark, the absorbance was read at 546 nm. Inhibition (%) was calculated as (Ablank − Asample)/Ablank × 100. The results were expressed as IC50 in µg quercetin equivalents per g dry weight (µg QE/g d.w.).

All *in vitro* measurements were performed in at least three independent experiments and are reported as mean ± SD. Spectrophotometric readings were obtained using a UV-Vis spectrophotometer (Jasco V-350, Jasco International Co., Ltd., Tokyo, Japan).

### 4.5. In Vivo Experimental Design

#### 4.5.1. Experimental Protocol

Adult male Wistar rats (200–250 g) obtained from the Establishment for Breeding and Use of Laboratory Animals, Iuliu Hațieganu University of Medicine and Pharmacy (Cluj-Napoca, Romania), were maintained under standard conditions (25 ± 1 °C; 55 ± 5% relative humidity; 12/12 h light/dark cycle) with ad libitum access to chow and water. Rats were randomized into 10 groups (*n* = 9). Lyophilized OSEE was administered by oral gavage at 1 g/kg/day (R100), 0.50 g/kg/day (R50), or 0.25 g/kg/day (R25), diluted in 1 mL dH_2_O/animal, prepared fresh daily. The SHAM group served as a healthy control without treatment. For the therapeutic protocol (six groups), acute inflammation was induced on day 1 by intramuscular turpentine oil (6 mL/kg b.w.) injection [39]. Treatments were then administered for 10 days: INFL1 (tap water), DICLO (diclofenac 10 mg/kg b.w./day) [40], TX (Trolox 50 mg/kg b.w./day) [41], or OSEE (R100/R50/R25). For the prophylactic protocol (five groups), treatments were then administered for 10 days—INFL11 (tap water), TX (Trolox 50 mg/kg b.w./day) [32], or OSEE (R100/R50/R25)—and acute inflammation was induced on day 11. On day 12, all rats were anesthetized with ketamine (60 mg/kg b.w.) and xylazine (15 mg/kg b.w.) [42]; blood was collected by retro-orbital puncture, serum was separated, and samples were stored at −80 °C until analysis.

The study was approved by the Cluj-Napoca Veterinary Sanitary Direction and Food Safety Committee (No. 372/4 July 2023). All procedures followed Directive 2010/63/EU and Romanian national law 43/2014 governing the protection of animals used for scientific purposes.

#### 4.5.2. Oxidative Stress Biomarker Assessment

##### Total Oxidative Status

Total oxidative status (TOS) was determined based on the oxidation of ferrous ions to ferric ions under acidic conditions in the presence of ROS. Absorbance was read at 560 nm, and values were expressed as µmol H_2_O_2_ equivalents/L (µmol H_2_O_2_ equiv/L) [43].

##### Total Antioxidant Capacity

Total antioxidant capacity (TAC) was measured by quantifying the inhibition of hydroxyl radical generation in a Fenton-type reaction by serum antioxidants. The absorbance was recorded at 444 nm, and the results were reported as mmol Trolox equivalents/L (mmol TE/L) [43].

##### Oxidative Stress Index

The oxidative stress index (OSI) was calculated as the oxidant-to-antioxidant ratio: OSI (arbitrary units) = TOS (mM H_2_O_2_ equiv/L)/TAC (mmol Trolox equiv/L). This provides an integrated estimate of systemic oxidative stress [43].

##### Advanced Oxidation Protein Products

Advanced oxidation protein products (AOPPs) were quantified spectrophotometrically as markers of protein oxidation [44]. Samples and chloramine-T standards were diluted to 10% in PBS, potassium iodide and glacial acetic acid were added, and the absorbance was measured at 340 nm. The concentrations were expressed as µmol chloramine-T equivalents/L.

##### Malondialdehyde

Malondialdehyde (MDA), as an index of lipid peroxidation, was measured using a thiobarbituric acid-reactive substances method [45]. Serum (0.1 mL) was mixed with 0.1 mL 40% trichloroacetic acid and 0.2 mL 0.67% thiobarbituric acid, heated for 30 min in a boiling water bath, cooled on ice, and centrifuged for 5 min at 3461 g. The absorbance at 532 nm was used to calculate the MDA concentration, reported as nmol/mL serum.

##### Nitric Oxide Synthesis

Nitric oxide production was estimated as total nitrites and nitrates (NOx) using the Griess reaction [46]. Serum proteins were removed by extraction with methanol/diethyl ether (3:1, *v*/*v*), nitrates were reduced to nitrites using vanadium (III) chloride, and the absorbance was read at 540 nm. The results were expressed as µmol/L.

##### 3-Nitrotyrosine

3-Nitrotyrosine (3-NT), a marker of peroxynitrite-mediated oxidative damage, was quantified by ELISA (E-EL-0040) according to the manufacturer’s instructions and reported as ng/mL.

##### Total Thiols

Total thiols (SH) were measured using Ellman’s reagent [47]. The absorbance was read at 412 nm, and the concentrations were expressed as mM glutathione equivalents per mL (mmol GSH/mL).

#### 4.5.3. Inflammatory Biomarker Assessment

Systemic inflammation was evaluated by ELISA measurement of NF-κB-p65, IL-1β, IL-18, caspase-1, and IL-10. The assays were conducted according to the kit protocols, and NF-κB-p65 results were expressed as ng/mL, and the IL-1β, IL-18, caspase-1, and IL-10 results were expressed as pg/mL.

Spectrophotometric assays were performed on a UV–Vis spectrophotometer (Jasco V-350, Jasco International Co., Ltd., Tokyo, Japan). ELISA procedures were performed using a Biotek Microplate 50 TS washer and an 800 TS microplate reader (Agilent Technologies Inc., Santa Clara, CA, USA).

### 4.6. Statistical Analysis

Results are presented as mean ± standard deviation (SD) for variables exhibiting a normal distribution. The normality of distribution for each continuous variable was assessed using the Kolmogorov–Smirnov test. Homogeneity of variances across groups was evaluated using Levene’s test. Group comparisons were carried out by one-way ANOVA followed by Tukey’s post hoc test. Pearson correlation and PCA were used to explore correlations between oxidative stress and inflammatory markers. Statistical significance was set at *p* < 0.05. Analyses were performed in SPSS Statistics v26.0 (SPSS, Chicago, IL, USA).

## 5. Conclusions

The whole-grain rice flour ethanol extract contained multiple polyphenols, with ferulic acid and *p*-coumaric acid among the most abundant, and showed both antioxidant and anti-inflammatory activity. In turpentine-induced acute inflammation, OSEE improved redox status by lowering oxidant and damage markers (TOS, OSI, AOPP, MDA, NOx, and 3-NT) and by restoring antioxidant measures (TAC and SH) under both therapeutic and prophylactic regimens. In parallel, OSEE reduced the expression of key pro-inflammatory mediators (NF-κB, IL-1β, IL-18, and caspase-1). These findings support further investigation of OSEE as a preventive or adjunct approach for conditions characterized by oxidative stress and inflammation, including long-term safety studies and mechanistic work to clarify molecular targets and translational relevance.

## Figures and Tables

**Table 1:** Tentative liquid chromatography–diode array detection–electro-spray ionization mass spectrometry identification of the phenolic compounds in whole-grain *Oryza sativa* L. flour ethanol extract.

Peak No.	R_t_ (min)	UV λ_max_ (nm)	[M + H]^+^ (*m*/*z*)	Compound	Subclass	Concentration (μg/mL)	References
1	3.05	280	155	2,3-Dihydroxybenzoic acid	Hydroxybenzoic acid	11.13 ± 0.21	[9]
2	3.39	280	139	2-Hydroxybenzoic acid	Hydroxybenzoic acid	5.21 ± 0.04	[10]
3	3.72	280	139	3-Hydroxybenzoic acid	Hydroxybenzoic acid	6.91 ± 0.55	[10]
4	5.09	275	171	Gallic acid	Hydroxybenzoic acid	13.21 ± 0.21	[11]
5	9.30	280	155	Protocatechuic acid	Hydroxybenzoic acid	18.64 ± 0.10	[11]
6	10.45	280	155	2,4-Dihydroxybenzoic acid	Hydroxybenzoic acid	4.68 ± 0.08	[9]
7	11.10	280	139	*p*-Hydroxybenzoic acid	Hydroxybenzoic acid	4.31 ± 0.19	[11]
8	11.95	330	355	Chlorogenic acid	Hydroxycinnamic acid	3.97 ± 0.13	[11]
9	12.50	330	181	Caffeic acid	Hydroxycinnamic acid	3.31 ± 0.13	[11]
10	12.97	340,250	449,287	Luteolin-glucoside	Flavone	13.94 ± 0.10	[12]
11	13.44	280	169	Vanillic acid	Hydroxybenzoic acid	2.14 ± 0.10	[10]
12	13.69	340,260	433,271	Apigenin-glucoside	Flavone	0.85 ± 0.07	[12]
13	14.05	340,260	565,271	Apigenin-glucoside-arabinoside	Flavone	0.98 ± 0.05	[12]
14	15.05	310	165	*p*-coumaric acid	Hydroxycinnamic acid	5.54 ± 0.09	[10]
15	15.88	331	195	Ferulic acid	Hydroxycinnamic acid	23.17 ± 0.28	[10]
16	18.40	280	199	Syringic acid	Hydroxybenzoic acid	7.09 ± 0.04	[10]
				Total phenolics		125.08 ± 0.05	

Values are means ± SD (*n* = 3) of three technical replicates.

**Table 3:** The *in vivo* therapeutic effect of whole-grain *Oryza sativa* L. flour ethanol extract on global oxidative stress markers.

	TOS (µmol H_2_O_2_ E/L)		TAC (mmol TE/L)		OSI	
CONTROL	5.70	±0.60	1.086	±0.0007	5.50	±0.44
INFL1	16.16	±1.84 ^aaa^	1.084	±0.0010 ^aaa^	16.23	±1.85 ^aaa^
INFL/DICLO	9.62	±1.05 *	1.087	±0.0007 ***	8.57	±1.09 ***
INFL/TX	10.36	±1.09 **	1.086	±0.0009 ***	9.55	±1.16 **
INFL/R100	11.85	±2.06 **	1.085	±0.0002 ***	10.41	±2.25 **
INFL/R50	9.98	±0.91 **	1.085	±0.0004 **	9.15	±1.95 **
INFL/R25	11.56	±1.82 **	1.084	±0.0004 **	10.44	±1.84 **

Values are means ± SD; ^a^ vs. CONTROL: ^aaa^ *p* < 0.001; * vs. INFL: * *p* < 0.05; ** *p* < 0.01; *** *p* < 0.001; TOS—total oxidative status; TAC—total antioxidant capacity; OSI—oxidative stress index; INFL1—inflammation induced by turpentine oil in the first day; DICLO—diclofenac (10 mg/kg); TX—Trolox (50 mg/kg); R100—whole-grain *Oryza sativa* L. flour ethanol extract 1 g/kg/day; R50—whole-grain *Oryza sativa* L. flour ethanol extract 0.50 g/kg/day; R25—whole-grain *Oryza sativa* L. flour ethanol extract 0.25 g/kg/day.

**Table 4:** The *in vivo* therapeutic effect of whole-grain *Oryza sativa* L. flour ethanol extract on specific oxidative stress markers.

	MDA (nmol/L)		AOPP (µmol/L)		NO (µmol/L)		3-NT (ng/mL)		SH (µmol/L)	
CONTROL	4.84	±0.37	81.36	±5.17	30.52	±3.96	26.00	±1.84	582.00	±55.97
INFL1	6.59	±0.44	140.51	±14.80 ^aaa^	79.79	±2.79 ^aaa^	55.95	±7.48 ^aaa^	317.40	±34.70 ^aaa^
INFL/DICLO	5.08	±0.42 ***	95.22	±10.23 ***	51.78	±1.06 ***	22.89	±3.92 ***	373.40	±25.08
INFL/TX	5.56	±0.14 ***	92.78	±9.87 ***	54.21	±8.70 ***	23.68	±4.16 ***	641.33	±54.06 ***
INFL/R100	5.92	±0.08 ***	82.96	±9.69 ***	48.78	±3.30 ***	21.90	±1.62 ***	530.33	±57.21 ***
INFL/R50	5.97	±0.20 ***	74.18	±8.48 ***	55.06	±6.15 **	33.85	±3.11 **	487.67	±31.78 **
INFL/R25	6.38	±0.30	64.14	±6.13 ***	44.53	±3.23 ***	15.59	±3.23 ***	413.00	±43.75 **

Values are means ± SD; ^a^ vs. CONTROL: ^aaa^ *p* < 0.001; * vs. INFL: ** *p* < 0.01; *** *p* < 0.001; MDA—malondialdehyde; AOPP—advanced oxidation protein products; NO—nitric oxide; 3-NT—3-nitrotyrosine; SH—total thiols; INFL1—inflammation induced by turpentine oil in the first day; DICLO—diclofenac (10 mg/kg); TX—Trolox (50 mg/kg); R100—whole-grain *Oryza sativa* L. flour ethanol extract 1 g/kg/day; R50—whole-grain *Oryza sativa* L. flour ethanol extract 0.50 g/kg/day; R25—whole-grain *Oryza sativa* L. flour ethanol extract 0.25 g/kg/day.

**Table 5:** The *in vivo* therapeutic effect of whole-grain *Oryza sativa* L. flour ethanol extract on inflammatory markers.

	NfkB-p65 (ng/mL)		IL-1b (pg/mL)		IL-18 (pg/mL)		Caspase 1 (pg/mL)		IL-10 (pg/mL)	
CONTROL	139.72	±15.92	26.37	±1.85	8.03	±0.64	51.40	±3.63	61.24	±4.44
INFL1	589.83	±43.19 ^aaa^	64.52	±2.70 ^aaa^	22.61	±2.84 ^aaa^	252.07	±15.69 ^aaa^	65.21	±5.55
INFL/DICLO	157.73	±15.19 ***	33.88	±2.29 ***	12.95	±1.16 **	12.85	±1.90 ***	65.65	±2.99
INFL/TX	155.06	±14.42 ***	34.97	±4.29 ***	8.52	±0.17 ***	65.09	±5.83 ***	63.74	±4.46
INFL/R100	61.75	±4.29 ***	42.34	±4.45 **	9.39	±0.55 ***	24.43	±2.05 ***	63.15	±5.11
INFL/R50	199.32	±11.96 ***	40.83	±3.23 **	11.14	±0.73 **	32.66	±2.60 ***	67.12	±5.37
INFL/R25	353.44	±16.67 ***	50.59	±4.57 *	13.18	±1.51 **	49.41	±2.36 ***	71.09	±5.79

Values are means ± SD; ^a^ vs. CONTROL: ^aaa^ *p* < 0.001; * vs. INFL: * *p* < 0.05; ** *p* < 0.01; *** *p* < 0.001; IL-1B—interleukin 1 beta; IL-10—interleukin 10; IL-18—interleukin 18; NFκB—nuclear factor kappa B; INFL1—inflammation induced by turpentine oil in the first day; TX—Trolox (50 mg/kg); R100—whole-grain *Oryza sativa* L. flour ethanol extract 1 g/kg/day; R50—whole-grain *Oryza sativa* L. flour ethanol extract 0.50 g/kg/day; R25—whole-grain *Oryza sativa* L. flour ethanol extract 0.25 g/kg/day.

**Table 6:** The *in vivo* prophylactic effect of whole-grain *Oryza sativa* L. flour ethanol extract on global oxidative stress markers.

	TOS (µmol H_2_O_2_ E/L)		TAC (mmol TE/L)		OSI	
CONTROL	5.70	±0.60	1.086	±0.0007	5.50	±0.44
INFL11	12.09	±1.92 ^aaa^	1.082	±0.0011 ^aaa^	12.01	±1.49 ^aaa^
TX/INFL	7.22	±0.82 **	1.087	±0.0007 **	7.08	±0.69 **
R100/INFL	6.02	±0.79 ***	1.086	±0.0004 **	5.44	±0.78 ***
R50/INFL	7.17	±0.22 **	1.085	±0.0003 **	6.31	±0.95 ***
R25/INFL	9.25	±1.93 *	1.085	±0.0004 **	7.94	±0.56 **

Values are means ± SD; ^a^ vs. CONTROL: ^aaa^ *p* < 0.001; * vs. INFL: * *p* < 0.05; ** *p* < 0.01; *** *p* < 0.001; TOS -total oxidative status; TAC—total antioxidant capacity; OSI—oxidative stress index; INFL11—inflammation induced by turpentine oil in the 11th day; TX—Trolox (50 mg/kg); R100—whole-grain *Oryza sativa* L. flour ethanol extract 1 g/kg/day; R50—whole-grain *Oryza sativa* L. flour ethanol extract 0.50 g/kg/day; R25—whole-grain *Oryza sativa* L. flour ethanol extract 0.25 g/kg/day.

**Table 7:** The *in vivo* prophylactic effect of whole-grain *Oryza sativa* L. flour ethanol extract on specific oxidative stress markers.

	MDA (nmol/L)		AOPP (µmol/L)		NO (µmol/L)		3-NT (ng/mL)		SH (µmol/L)	
CONTROL	4.84	±0.37	81.36	±5.17	30.52	±3.96	26.00	±1.84	582.00	±55.97
INFL11	6.78	±0.44 ^aaa^	120.62	±8.72 ^aaa^	66.15	±4.63 ^aaa^	42.21	±3.52 ^aa^	390.26	±24.09 ^aaa^
TX/INFL	5.64	±0.31 ***	86.55	±7.92 **	55.23	±4.89 **	39.47	±3.41	522.03	±44.24 **
R100/INFL	5.49	±0.41 ***	71.21	±6.35 **	49.71	±3.09 **	21.96	±1.56 **	537.00	±51.04 **
R50/INFL	5.48	±0.31 ***	55.71	±2.34 ***	50.99	±5.21 **	22.89	±1.34 **	423.67	±33.36 *
R25/INFL	5.74	±0.34 ***	79.54	±3.36 **	56.62	±4.77 **	13.53	±1.32 ***	561.00	±54.33 **

Values are means ± SD; ^a^ vs. CONTROL: ^aa^ *p* < 0.01; ^aaa^ *p* < 0.001; * vs. INFL: * *p* < 0.05; ** *p* < 0.01; *** *p* < 0.001; MDA—malondialdehyde; AOPP—advanced oxidation protein products; NO—nitric oxide; 3-NT—3-nitrotyrosine; SH—total thiols; INFL11—inflammation induced by turpentine oil in the 11th day; TX—Trolox (50 mg/kg); R100—whole-grain *Oryza sativa* L. flour ethanol extract 1 g/kg/day; R50—whole-grain *Oryza sativa* L. flour ethanol extract 0.50 g/kg/day; R25—whole-grain *Oryza sativa* L. flour ethanol extract 0.25 g/kg/day.

**Table 8:** The *in vivo* prophylactic effect of whole-grain *Oryza sativa* L. flour ethanol extract on inflammatory markers.

	NfkB-p65 (ng/mL)		IL-1β (pg/mL)		IL-18 (pg/mL)		Caspase-1 (pg/mL)		IL-10 (pg/mL)	
CONTROL	139.72	±15.92	26.37	±1.85	8.03	±0.64	51.40	±3.63	61.24	±4.44
INFL11	389.27	±28.12 ^aaa^	60.05	±4.25 ^aaa^	42.26	±3.41 ^aaa^	196.34	±17.33 ^aaa^	54.98	±4.68
TX/INFL	153.06	±9.52 ***	29.38	±6.25 ***	8.65	±1.61 ***	48.26	±3.49 ***	55.32	±2.45
R100/INFL	241.06	±2.18 ***	47.05	±3.55	9.85	±0.64 ***	45.11	±4.12 ***	48.59	±3.11
R50/INFL	270.91	±8.86 ***	45.67	±3.83 **	6.70	±0.41 ***	48.87	±2.30 ***	59.32	±1.69
R25/INFL	283.81	±6.01 ***	37.64	±2.83 ***	5.91	±0.88 ***	99.03	±4.11 ***	56.43	±2.00

Values are means ± SD (*n* = 9); ^a^ vs. CONTROL: ^aaa^ *p* < 0.001; * vs. INFL: ** *p* < 0.01; *** *p* < 0.001; IL-1B—interleukin 1 beta; IL-10—interleukin 10; IL-18—interleukin 18; NFκB—nuclear factor kappa B; INFL11—inflammation induced by turpentine oil in the 11th day; TX—Trolox (50 mg/kg); R100—whole-grain *Oryza sativa* L. flour ethanol extract 1 g/kg/day; R50—whole-grain *Oryza sativa* L. flour ethanol extract 0.50 g/kg/day; R25—whole-grain *Oryza sativa* L. flour ethanol extract 0.25 g/kg/day.

## Data Availability

The data will be available in the PhD thesis of the first author after the thesis defense at “Iuliu Hațieganu” University of Medicine and Pharmacy Cluj-Napoca.
